# Summarized data of genotoxicity tests for designated food additives in Japan

**DOI:** 10.1186/s41021-018-0115-2

**Published:** 2018-12-26

**Authors:** Masami Yamada, Masamitsu Honma

**Affiliations:** 1 Department of Applied Chemistry, National Defense Academy, 1-10-20, Hashirimizu, Yokosuka-shi, Kanagawa 239-8686 Japan; 2 Division of Genetics and Mutagenesis, National Institute of Health Sciences, 3-25-26, Tonomachi, Kawasaki-ku, Kawasaki-shi, Kanagawa 210-9501 Japan

**Keywords:** Food additives, Designated additives, Genotoxicity test, Ames test, Transgenic rodent gene mutation assay

## Abstract

The Ministry of Health, Labour and Welfare has carried out genotoxicity tests for food additives used in Japan in cooperation with the Japan Food Additives Association since 1979. Hayashi et al. summarized these data and published a list of 337 designated additives (Shitei-tenkabutsu in Japanese) with genotoxicity test data in 2000. Thereafter, 29 items were eliminated, and 146 items were newly added. Currently, 454 designated additives are allowed to be used as food additives in Japan. This report, based on the Hayashi report, covers the addition of newly derived genotoxicity test data. Routinely, the bacterial reverse mutation test (Ames test), mammalian cell chromosomal aberration test, and in vivo rodent bone marrow micronucleus test have been used for the evaluation of genotoxicity of food additives. In addition to the data from these tests being updated in this report, it newly includes results of transgenic rodent somatic and germ cell gene mutation assays (TGR assays), incorporated in the Organisation for Economic Co-operation and Development (OECD) test guidelines after 2000. We re-evaluated the genotoxicity of 13 designated food additives considering their TGR data.

**Electronic supplementary material:**

The online version of this article (10.1186/s41021-018-0115-2) contains supplementary material, which is available to authorized users.

## Background

Since 1979, as part of the safety reassessment of food additives, the Ministry of Health, Labour and Welfare (MHLW; prior to January 2001, the Ministry of Health and Welfare) has carried out mutagenicity tests annually in cooperation with the Japan Food Additives Association. In 2000, Dr. M. Hayashi (former Head of Division of Genetics and Mutagenesis at the National Institute of Health Sciences (NIHS)) and colleagues summarized the mutagenicity data for 337 designated additives, 187 existing additives (natural additives), 49 natural fragrances, and seven general food and drink additives from fiscal year (FY) 1979 to FY1998 [1] (hereafter referred to as the “Hayashi report”). Since then, concerning designated additives, 29 items have been eliminated due to abolition of form classification or for other reasons (Table 1), and 146 items have been newly added (Table 2). In this report, which is based on the Hayashi report, data on newly tested items have been added, and mutagenicity data for a total of 454 designated food additives is summarized in Table 3.

**Table 1:** List of designated food additives eliminated after 2000 (As of October 6, 2016)

Name	Date	Reason
Aluminum Potassium Sulfate (dried) (syn: Burnt Alum)	June 30, 2000	Integrated into “Aluminum Potassium Sulfate”
Ferrous Pyrophosphate	June 30, 2000	Distribution and usage records have not been confirmed
Sodium Sulfite (anhydrous)	June 30, 2000	Integrated into “Sodium Sulfite”
Tetrasodium Pyrophosphate (anhydrous)	June 30, 2000	Integrated into “Tetrasodium Pyrophosphate”
Aluminum Ammonium Sulfate (dried) (syn: Burnt Ammonium Alum)	June 30, 2000	Integrated into “Aluminum Ammonium Sulfate”
Disodium Hydrogen Phosphate (anhydrous) (Disodium Phosphate (anhydrous))	June 30, 2000	Integrated into “Disodium Hydrogen Phosphate”
Sodium Dihydrogen Phosphate (anhydrous) (Monosodium Phosphate (anhydrous))	June 30, 2000	Integrated into “Sodium Dihydrogen Phosphate”
Tertiary Sodium Phosphate (anhydrous)	June 30, 2000	Integrated into “Tertiary Sodium Phosphate”
Choline phosphate	June 30, 2000	Distribution and usage records have not been confirmed
Methyl *O*-Acetylricinoleate	June 30, 2000	Distribution and usage records have not been confirmed
Citric Acid (anhydrous)	June 30, 2000	Integrated into “Citric Acid”
Ferrous Sulfate (dried)	June 30, 2000	Integrated into “Ferrous Sulfate”
Sodium Acetate (anhydrous)	June 30, 2000	Integrated into “Sodium Acetate”
Sodium Hydroxide (crystal)	June 30, 2000	Integrated into “Sodium Hydroxide”
Sodium Carbonate (crystal)	June 30, 2000	Integrated into “Sodium Carbonate”
Sodium Starch Phosphate	June 4, 2009	Production and usage have not been confirmed

The date and the reasons for disappearance are indicated

**Table 2:** List of items that have been added to the designated food additives (As of October 6, 2016)

No.^a^	Name
2	chlorous acid water
4	nitrous oxide
8	calcium l-ascorbate
9	l-ascorbic acid 2-glucoside
13	asparaginase
16	acesulfame potassium
17	acetylated distarch adipate
18	acetylated oxidized starch
19	acetylated distarch phosphate
20	acetaldehyde
24	sodium selenite pentahydrate
25	azoxystrobin
26	advantame
28	β-apo-8′-carotenal
29	(3-amino-3-carboxypropyl) dimethylsulfonium chloride
30	amylalcohol
35	ammonium alginate
36	potassium alginate
37	calcium alginate
44	ammonium isovalerate
46	ion exchange resin
47	isoamylalcohol
51	isoquinoline
54	isovaleraldehyde
55	isobutanol
56	isobutyraldehyde (isobutanal)
57	isopropanol
58	isopentylamine
67	mixture of 2-ethyl-3,5-dimethylpyrazine and 2-ethyl-3,6-dimethylpyrazine
69	2-ethylpyrazine
70	3-ethylpyridine
71	2-ethyl-3-methylpyrazine
72	2-ethyl-5-methylpyrazine
73	2-ethyl-6-methylpyrazine
74	5-ethyl-2-methylpyridine
77	ethers
89	octanoic acid
91	starch sodium octenyl succinate
94	peracetic acid
102	canthaxanthin
106	xylitol (alias xylit)
110	triethyl citrate
113	sodium ferrous citrate (sodium iron citrate)
127	sodium gluconate
128	glutamyl-valyl-glycine
130	monoammonium l-glutamate
132	monocalcium *dl*- l -glutamate
134	monomagnesium *dl*-l-glutamate
135	calcium silicate
136	magnesium silicate
140	ketones
150	calcium acetate
156	starch acetate
165	calcium saccharin
168	calcium oxide
169	oxidized starch
172	hypochlorous acid water
174	hypobromous acid water
176	2,3-diethylpyrazine
177	2,3-diethyl-5-methylpyrazine
189	fatty acids
191	aliphatic higher aldehydes (except those generally recognized as highly toxic)
192	aliphatic higher hydrocarbons (except those generally recognized as highly toxic)
193	2,3-dimethylpyrazine
194	2,5-dimethylpyrazine
195	2,6-dimethylpyrazine
196	2,6-dimethylpyridine
201	potassium *dl*-bitartrate (potassium hydrogen *dl*-tartrate or potassium hydrogen *dl*-tartrate)
203	disodium *dl*-tartrate (disodium *dl*-tartrate)
226	magnesium hydroxide
227	sucralose (trichlorogalactosucrose)
228	calcium stearate
229	magnesium stearate
231	sodium stearoyl lactylate
236	calcium sorbate
248	thiamine thiocyanate (vitamin B1 thiocyanate)
251	thioethers (except those generally recognized as highly toxic)
252	thiols (thioalcohols) (except those generally recognized as highly toxic)
258	5,6,7,8-tetrahydroquinoxaline
259	2,3,5,6-tetramethylpyrazine
262	terpene hydrocarbons
268	all-racemic -α-tocopheryl acetate
269	R,R,R -α-tocopheryl acetate
272	trimethylamine
273	2,3,5-trimethylpyrazine
276	nisin
277	natamycin
284	carbon dioxide (carbonic acid, gas)
287	potassium lactate
291	neotame
303	valeraldehyde
306	biotin
308	bisbentiamine (benzoylthiamine disulfide)
311	1-hydroxyethylidene-1, 1-diphosphonic acid
313	hydroxycitronellal dimethylacetal
314	hydroxypropyl distarch phosphate
315	hydroxypropyl cellulose
316	hydroxypropyl starch
317	hydroxypropyl methylcellulose
318	piperidine
321	sunflower lecithin
323	pyrazine
325	pyrimethanil
328	pyrrolidine
334	pyrrole
339	2-(3-phenylpropyl) pyridine
340	phenethylamine
341	phenol ethers (except those generally recognized as highly toxic)
342	phenols (except those generally recognized as highly toxic)
343	ferrocyanides (potassium ferrocyanide (potassium hexacyanoferrate (ii)), calcium ferrocyanide (calcium hexacyanoferrate (ii)), sodium ferrocyanide (sodium hexacyanoferrate (ii))
344	butanol
345	butylamine
346	butyraldehyde
350	fludioxonil
352	propanol
353	propionaldehyde
369	2-pentanol (syn: *sec*-amylalcohol)
370	trans-2-pentenal
371	1-penten-3-ol
372	aromatic alcohols
373	aromatic aldehydes (except those generally recognized as highly toxic)
377	polysorbate 20
378	polysorbate 60
379	polysorbate 65
380	polysorbate 80
381	polyvinylpyrroridone
382	polyvinylpolypyrrolidone
394	5-methylquinoxaline
395	6-methylquinoline
396	6,7-dihydro-5-methyl-5 h-cyclopentapyrazine
398	1-methylnaphthalene
400	2-methypyrazine
401	2-methylbutanol
402	3-methyl-2-butanol
403	2-methylbutyraldehyde
404	trans-2-methyl-2-butenal, (*e*)-2-methyl-2-butenal
405	3-methyl-2-butenal
406	3-methyl-2-butenol
417	Lactones (except those generally recognized as highly toxic)
422	calcium 5′-ribonucleotide
431	potassium sulfate
439	distarch phosphate
440	monostarch phosphate
443	trimagnesium phosphate (syn: Magnesium phosphate, tribasic)
452	magnesium monohydrogen phosphate
454	phosphated distarch phosphate

^a^numbers are consistent with those underlined in Table 3

**Table 3:** List of the results in genotoxicity tests for the 454 designated food additives (As of October 6, 2016)

No.^a^	Name	Functional classes	CAS#	Molecular weight	Genotoxicity tests					Remarks
					Ames	CA	MN	TGR	Others	
1	zinc salts (limited to zinc gluconate and zinc sulfate)	Dietary supplement			-^(13)^	-^H23 c^				
**2** ^b^	chlorous acid water	Sterilizer	13898–47-0	68.45	+^FSC1 d^	+^FSC1^	-^FSC1^			
3 [20]	sodium chlorite	Bleaching agent etc.	7758-19-2	90.44	+^(2)^-^(12)^	+^(2)^	+^(9)^			
**4**	nitrous oxide	Propellantt	10024–97-2	44.01	-^FSC2^					
5	adipic acid	Acidifier	124–04-9	146.14	-^(15)^	-^H22^				
6	sodium nitrite	Color fixative	7632–00-0	69.00	+^(1)^ -^(16)^	+^(1)^	-^(9)^	-^H21^		Target organs for TG in mice: liver, stomach (glandular stomach)
7	l-ascorbic acid (vitamin C)	Antioxidant etc.	50–81-7	176.12	-^(1)(10)^	-^(1)^				
**8**	calcium l -ascorbate	Dietary supplement etc.	5743-28-2	426.35	-^FSC3^					
**9**	l-ascorbic acid 2-glucoside	Antioxidant etc.	129499–78-1	338.26	-^H26^	-^H26^	-^FSC4^			
10	l-ascorbic stearate (vitamin C stearate)	Antioxidant etc.	25395–66-8	442.59	-^(4)(16)^	-^(4)^				
11	sodium l-ascorbate (vitamin C sodium)	Antioxidant etc.	134–03-2	198.11	-^H20^	-^H20^	-^H20^			
12	l-ascorbic palmitate (vitamim C palmitate)	Antioxidant etc.	137–66-6	414.53	-^(17)^	-^H22^				
**13**	asparaginase	Processing agent	9015–68-3		-^FSC5^	-^FSC5^				
14	monosodium l -aspartate	Seasoning etc.	3792-50-5	173.10	-^(3)(17)^	-^(3)^				
15	aspartame (α- l -aspartyl- l -phenylalanine methyl ester)	Sweetener	22839–47-0	294.30	-^(16)^	-^H23^				
**16** [21, 22]	acesulfame potassium	Sweetener etc.	55589–62-3	201.24	*-* ^(21)^	*-* ^(22)^				
**17**	acetylated distarch adipate	Thickening agents etc.	68130–14-3							Evaluated as modified starch
**18**	acetylated oxidized starch	Thickening agents etc.	68187–08-6							Evaluated as modified starch
**19**	acetylated distarch phosphate	Thickening agents etc.	–							Evaluated as modified starch
**20**	acetaldehyde	Flavoring agent etc.	75–07-0	44.05	-^FSC6^	+^FSC6^				
21	ethyl acetoacetate	Flavoring agent	141–97-9	130.14	-^(3)(12)^	-^(3)^				
22	acetophenone	Flavoring agent	98–86-2	120.15	-^(12)^					
23	acetone	Processing agent	67–64-1	58.08	-^(1)(17)^	+^(1)^				
**24**	sodium selenite pentahydrate	Dietary supplement	10102–18-8	172.94	+^FSC7^	+^FSC7^	+^FSC7^			
**25**	azoxystrobin	Preservative etc.	131860–33-8	403.4	-^FSC8^		-^FSC8^			
**26**	advantame	Sweetener	714229–20-6	476.52	-^FSC9^		-^FSC9^			
27	anisaldehyde (p-methoxybenzaldehyde)	Flavoring agent	123–11-5	136.15	-^(2)(12)^	-^(2)^				
**28**	β-apo-8′-carotenal	Food color	1107–26-2	416.64	-^FSC10^	-^FSC10^	-^FSC10^			
**29**	(3-amino-3-carboxypropyl) dimethylsulfonium chloride	Flavoring agent	3493-12-7	199.7	-^H21^	-^H21^	-^H21^			
**30**	amylalcohol	Flavoring agent	71–41-0	88.15	*-* ^H16^	*-* ^H16^				
31	α-amylcinnamaldehyde (α-amylcinnamic aldehyde)	Flavoring agent	122–40-7	202.29	-^(12)^	-^H24^				
32	dl-alanine	Seasoning etc.	302–72-7	89.09	-^(3)(17)^	-^(3)^				
33	sodium sulfite	Preservative etc.	7757-83-7	126.04	-^(1)(12)^	-^(1)^				Crystalline form and anhydrous form were used for tests in (1) and (12), respectively.
34	l -arginine l -glutamate	Seasoning etc.	4320–30-3	321.33	-^(4)(20)^	-^(4)^				
**35**	ammonium alginate	Emulsifier etc.	9005–34-9		-^FSC11^					
**36**	potassium alginate	Emulsifier etc.	9005–36-1		-^FSC12^					
**37**	calcium alginate	Emulsifier etc.	9005–35-0							See compounds with different salt
38 [23]	sodium alginate	Thickening agents	9005–38-3		-^(2)(8)(18)^	-^(2)^				
39	propylene glycol alginate	Thickening agents			-^(2)(13)^	-^(2)^				
40	benzoic acid	Preservative	65–85-0	122.12	-^(2)(11)^	-^(2)^				
41	sodium benzoate	Preservative	532–32-1	144.10	-^(1)(19)^	+^(1)^	-^H20^			
42	methyl anthranilate	Flavoring agent	134–20-3	151.16	-^(12)^	+^H22^				
43	ammonia	Processing agent	7664-41-7	17.03	-^(18)^					
**44**	ammonium isovalerate	Flavoring agent	1449430–58-3	323.43	-^H17^	-^H17^				
45	ionone	Flavoring agent	8013-90-9	192.30	-^(20)^	-^H24^				Mixture of α and β-ionone were used for the assay
**46**	ion exchange resin	Processing agent								
**47**	isoamylalcohol	Flavoring agent	123–51-3	88.15	*-* ^H16^	*-* ^H16^	*-* ^H16^			
48	isoeugenol	Flavoring agent	97–54-1	164.20	-^(12)^	-^H23^				
49	isoamyl isovalerate	Flavoring agent	659–70-1	172.26	-^(3)(12)^	-^(3)^				
50	ethyl isovalerate	Flavoring agent	108–64-5	130.18	-^(3)(12)^	-^(3)^				
**51**	isoquinoline	Flavoring agent	119–65-3	129.16		+^H18^	-^H21^			
52 [24]	isothiocyanates (except those generally recognized as highly toxic)	Flavoring agent	542–85-8		-^(19)^					Ethyl thiocyanate was used for the assay.
53	allyl isothiocyanate (volatile oil of mustard)	Flavoring agent	57–06-7	99.16	-^(12)^	+^H22^				
**54**	isovaleraldehyde	Flavoring agent	590–86-3	86.13	-^FSC13^		*-* ^H17^			
**55**	isobutanol	Flavoring agent	78–83-1	74.12	-^FSC14^	*-* ^H16^				
**56**	isobutyraldehyde (isobutanal)	Flavoring agent	78–84-2	72.11	-^FSC15^	+^FSC15^	-^FSC15^			
**57**	isopropanol	Processing agent etc.	67–63-0	60.10	-^FSC16^		-^FSC16^			
**58**	isopentylamine	Flavoring agent	107–85-7	87.16	-^H18^	-^H18^	-^H18^			
59 [25]	l -isoleucine	Dietary supplement	73–32-5	131.17	-^(3)(15)^	-^(3)^				
60	disodium 5′-inosinate (sodium 5′-inosinate)	Seasoning etc.	4691–65-0	392.17	-^(1)(17)^	+^(1)^	-^H20^			
61	imazalil	Antimolding agent	35554–44-0	297.18	-^(20)^					
62	indoles and its derivatives	Flavoring agent	120–72-9 (indole)		-^(19)^-^H26^	-^H23^				
63	disodium 5′-uridylate (sodium 5′-uridylate)	Seasoning etc.	3387–36-8	368.15	-^(1)(17)^	+^(1)^	-^H22^			
64	γ-undecalactone (undecalactone)	Flavoring agent	104–67-6	184.28	-^(3)(12)^	-^(3)^	-^(9)^			
65	ester gum	Chewing gum base			-^(2)^	-^(2)^				
66	esters	Flavoring agent			-^(2) or (4)^	-^(2) or (4)^				Cinnamyl anthranilate, ethyl caprylate, allyl caproate, ethyl caproate were included.
**67**	mixture of 2-ethyl-3,5-dimethylpyrazine and 2-ethyl-3,6-dimethylpyrazine	Flavoring agent	55031–15-7	136.20	-^FSC17^					
68	ethylvanillin	Flavoring agent	121–32-4	166.17	-^(2)(12)^	-^(2)^				
**69**	2-ethylpyrazine	Flavoring agent	13925–00-3	108.14			+^H16^			
**70**	3-ethylpyridine	Flavoring agent	536–78-7	107.15		+^H16^	-^H17^			
**71**	2-ethyl-3-methylpyrazine	Flavoring agent	15707–23-0	122.17	*-* ^H16^	*-* ^H16^	*-* ^H16^			
**72**	2-ethyl-5-methylpyrazine	Flavoring agent	13360–64-0	122.17	*-* ^H16^	*-* ^H16^				
**73**	2-ethyl-6-methylpyrazine	Flavoring agent	13925–03-6	122.17	*-* ^H17^	*-* ^H17^				The test substrate was mixture with 2-ethyl-5-methylpyrazine.
**74**	5-ethyl-2-methylpyridine	Flavoring agent	104–90-5	121.18	-^FSC18^		-^FSC18^			
75	calcium disodium ethylenediaminetetraacetate (calcium disodium edta)	Antioxidant	62–33-9	410.30	-^(13)^	-^H22^				
76	disodium ethylenediaminetetraacetate (disodium, EDTA)	Antioxidant	6381-92-6	372.24	-^(13)^					
**77**	ethers	Flavoring agent								
78	erythorbic acid (isoascorbic acid)	Antioxidant	89–65-6	176.12	+^(2)^ -^(17)^	-^(2)^	-^(9)^	-^H21^		Target organs for TGR in mice:liver, stomach (glandular stomach)
79	sodium erythorbate (sodium isoascorbate)	Antioxidant	6381–77-7	216.12	-^(1)(14)^	-^(1)^				
80	ergocalciferol (calciferol or vitamin D2)	Dietary supplement	50–14-6	396.65	-^(1)(20)^	-^(1)^				
81	ammonium chloride	Raising agent	12125–02-9	53.49	-^(2)(16)^	+^(2)^	-^(9)^			
82	potassium chloride	Seasoning	7447-40-7	74.55	-^(17)^	-^H23^				
83	calcium chloride	Tofu coagulator etc.	10043–52-4	110.98	-^(1)(12)^	-^(1)^				
84	ferric chloride	Dietary supplement	10025–77-1	270.29	-^(4)(15)^	-^(4)^				
85	magnesium chloride	Tofu coagulator etc.	7791-18-6	203.30	-^(4)(5)(18)^	-^(4)^				
86	hydrochloric acid	Processing agent	7647–01-0	36.46	-^(16)^					
87	eugenol	Flavoring agent	97–53-0	164.20	-^(2)(14)^	+^(2)^	-^(9)^			
88	octanal (capryl aldehyde or octyl aldehyde)	Flavoring agent	124–13-0	128.21	-^(14)^	-^H22^				
**89**	octanoic acid	Flavoring agent	124–07-2		-^FSC19^					
90	ethyl octanoate (ethyl caprylate)	Flavoring agent	106–32-1	172.26	-^(14)^	-^H25^				
**91**	starch sodium octenyl succinate	Thickening agents etc.	–		-^FSC20^					
92	*o* -phenylphenol and sodium *o* -phenylphenate	Antimolding agent	90–43-7	170.21	-^(1)^					*o* -phenylphenol was negative in CA
93	sodium oleate	Film-forming agent	143–19-1	304.44	-^(14)^					
**94**	peracetic acid	Preservative etc.	79–21-0		+^FSC21^	+^FSC21^	-^FSC21^			
95	hydrogen peroxide	Sterilizer	7722-84-1	34.01	-^(1)(16)^	+^(1)^	-^H21^			
96	benzoyl peroxide	Flour treatment agent	94–36-0	242.23	-^(1)(12)^	-^(1)^				
97	sodium caseinate	Processing agent	9005-46-3		-^(5)(18)^	-^(5)^				
98	ammonium persulfate	Flour treatment agent	7727-54-0	228.20	-^(2)(12)^	-^(2)^				
99	calcium carboxymethylcellulose (calcium cellulose glycolate)	Thickening agents	9050-04-8		-^(4)^	-^(4)^				
100	sodium carboxymethylcellulose (sodium cellulose glycolate)	Thickening agents	9004-32-4		-^(1)(13)^	-^(1)^				
101	β-carotene	Food color etc.	7235-40-7	536.88	-^(1)(13)^	±^(1)^				
**102**	canthaxanthin	Food color	514–78-3	564.82	-^FSC22^	-^FSC22^	-^FSC22^			
103	isoamyl formate	Flavoring agent	110–45-2	116.16	-^(3)(16)^	-^(3)^				
104	geranyl formate	Flavoring agent	105–86-2	182.26	-^(14)^	-^H24^				
105	citronellyl formate	Flavoring agent	105–85-1		-^(20)^	-^H24^				
**106** [26]	xylitol	Sweetener	87–99-0	152.15	*-* ^(23)^		*-* ^(23)^			
107	disodium 5′-guanylate (sodium 5′-guanylate)	Flavoring agent etc.	5550-12-9	407.18	-^(3)(17)^	-^(3)^				
108	citric acid	Acidifier	77–92-9	192.12	-^(1)(15)^	-^(1)^				Crystalline form and anhydrous form were used for tests in (1) and (5), respectively.
109	isopropyl citrate	Antioxidant	39413–05-3		-^(13)^					
**110**	triethyl citrate	Sweetener	77–93-0	276.28	-^H26^	-^H26^	-^H26^			
111	monopotassium citrate and tripotassium citrate	Flavoring agent etc.	866–83-1	230.21	-^(17)^	-^(8)^-^H25^			Rec assay: +^(8)^	monopotassium citrate and tripotassium citrate were used in (17), monopotassium citrate was used in (8), tripotassium citrate was used in H25
112	calcium citrate	Dietary supplement etc.	813–94-5	570.49	-^(5)(13)^	-^(5)^				
**113**	sodium ferrous citrate (sodium iron citrate)	Dietary supplement	50717–86-7	526.01	-^H22^	+^H22^				
114	ferric citrate	Dietary supplement	77–92-9	192.12	-^(4)(15)^	-^(4)^				
115	ferric ammonium citrate	Dietary supplement	1185–57-5		-^(4)(15)^	-^(4)^				
116	trisodium citrate (sodium citrate)	Acidifier	68–04-2	258.07	-^(17)^	-^(1)^				
117	glycine	Seasoning etc.	56–40-6	75.07	-^(3)(19)^	-^(3)^				Crystalline form and powdery form were used for tests in (3), and Crystalline form for (19)
118	glycerol (glycerin)	Processing agent	56–81-5	92.09	-^(2)(19)^	-^(2)^				
119	glycerol esters of fatty acids	Emulsifier			-^(1)^	-^(1)^				
120	calcium glycerophosphate	Dietary supplement	27214–00-2	210.14	-^(2)(12)^	-^(2)^				
121	disodium glycyrrhizinate	Sweetener	68797–35-3	899.11	-^(1)^	+^(1)^	-^(9)^			
122	glucono-delta-lactone (gluconolactone)	Acidifier	90–80-2	178.14	-^(1)(15)^	-^(1)^				
123	gluconic acid	Acidifier	526–95-4	196.16	-^(4)(15)^	-^(4)^				
124	potassium gluconate	Acidifier	299–27-4	234.25		-^(8)^			Rec assay: -^(8)^	
125	calcium gluconate	Dietary supplement	299–28-5	448.39	-^(5)(12)^	-^(5)^				
126	ferrous gluconate (iron gluconate)	Dietary supplement etc.	299–29-6	446.14	-^(19)^	+^H25^				
**127**	sodium gluconate	Emulsifier etc.	527–07-1	218.14						See substrates with different salt
**128**	glutamyl-valyl-glycine	Seasoning	38837–70-6	303.31	-^FSC23^	-^FSC23^	-^FSC23^			
129	l-glutamic acid	Seasoning	56–86-0	147.13	-^(5)(19)^	-^(5)^				
**130**	monoammonium l-glutamate	Seasoning etc.	7558-63-6	182.18	-^FSC24^				Rec assay: -^FSC24^	
131	monopotassium l-glutamate	Seasoning etc.	6382-01-0	203.23	-^(17)^	-^H25^				
**132**	monocalcium di-l-glutamate	Dietary supplement etc.	69704–19-4	404.38						See substrate with different salt
133	monosodium l-glutamate	Seasoning etc.	6106-04-3	187.13	-^(1)(19)^	-^(1)^				
**134**	monomagnesium di- l-glutamate	Dietary supplement etc.	129160–51-6	388.61						See substrate with different salt
**135**	calcium silicate	Processing agent	38837–70-6		-^FSC25^	-^FSC25^				
**136**	magnesium silicate	Processing agent	1343-88-0		-^FSC26^					
137	cinnamic acid	Flavoring agent	140–10-3	148.16	-^(14)^	-^H23^				
138	ethyl cinnamate	Flavoring agent	103–36-6	176.21	-^(2)(14)^	-^(2)^				
139	methyl cinnamate	Flavoring agent	103–26-4	162.19	-^(14)^	+^H23^				
**140**	ketones	Flavoring agent								
141	geraniol	Flavoring agent	106–24-1	154.25	-^(2)(14)^	-^(2)^				
142	high test hypochlorite	bleaching agent etc.			+^(3)^ -^(20)^	+^(3)^	-^(4)^			
143	succinic acid	Acidifier etc.	110–15-6	118.09	-^(1)(15)^	-^(1)^				
144	monosodium succinate	Seasoning etc.	2922-54-5	140.07	-^(19)^	-^H23^-^H26^				
145	disodium succinate	Seasoning etc.	150–90-3	162.05	-^(1)(19)^	±^(1)^				
146	cholecalciferol (vitamin D3)	Dietary supplement	67–97-0	384.64	-^(3)(20)^	-^(3)^				
147	sodium chondroitin sulfate	Humectant etc.	12678–07-8		-^(3)(14)^	-^(3)^				
148	isoamyl acetate	Flavoring agent	123–92-2	130.18	-^(3)(14)^	-^(3)^				
149	ethyl acetate	Flavoring agent etc.	141–78-6	88.11	-^(1)(16)^	+^(1)^	-^(9)^			
**150**	calcium acetate	Dietary supplement etc.	62–54-4	158.17						See substrates with different salt
151	geranyl acetate	Flavoring agent	105–87-3	196.29	-^(14)^	-^H24^				
152	cyclohexyl acetate	Flavoring agent	622–45-7	142.20	-^(14)^	-^H25^				
153	citronellyl acetate	Flavoring agent	150–84-5	198.30	-^(14)^	-^H24^				
154	cinnamyl acetate	Flavoring agent	103–54-8	176.21	-^(14)^	+^H24^				
155	terpinyl acetate	Flavoring agent	8007-35-0	196.29	-^(14)^	-^H24^				
**156**	starch acetate	Thickening agents etc.	9045–28-7		-^FSC20^	-^FSC20^	-^FSC20^			
157	sodium acetate	Acidifier etc.	127–09-3	82.03	-^(2)(18)^	-^(2)^				Crystalline form was used in (2), and anhydrous form was used in (18).
158	polyvinyl acetate	Chewing gum base etc.			-^(3)(14)^	-^(3)^				
159	phenethyl acetate (phenylethyl acetate)	Flavoring agent	103–45-7	164.20	-^(15)^	-^H24^				
160	butyl acetate	Flavoring agent	123–86-4	116.16	-^(3)(15)^	-^(3)^				
161	benzyl acetate	Flavoring agent	140–11-4	150.17	-^(4)(15)^	-^(4)^				
162	l-menthyl acetate	Flavoring agent	2623-23-6	198.30	-^(15)^	-^H22^				
163	linalyl acetate	Flavoring agent	115–95-7	196.29	-^(15)^	-^H23^				
164	saccharin	Sweetener	81–07-2	183.19	-^(2)(14)^	-^(2)^				
**165**	calcium saccharin	Sweetener	6381-91-5	467.48		+^FSC27^				
166 [27]	sodium saccharin (soluble saccharin)	Sweetener	128–44-9	205.17	-^(1)(11)^	+^(1)^	-^H21^			
167	methyl salicylate	Flavoring agent	119–36-8	152.15	-^(2)(15)^	-^(2)^				
**168**	calcium oxide	Processing agent	1305-78-8	56.08	-^FSC28^					
**169**	oxidized starch	Thickening agents etc.	–		-^FSC20^	-^FSC20^	-^FSC20^			
170	magnesium oxide	Absorbent etc.	1309–48-4	40.30	-^(13)^	-^H22^				
171 [28]	iron sesquioxide (diiron trioxide or iron oxide red)	Food color	1309–37-1	159.69	-^(4)(6)(19)^	-^(4)^	-^(8)^		Rec assay: -^(6)^	
**172**	hypochlorous acid water	Preservative			*-* ^H6^					
173	sodium hypochlorite (hypochlorite of soda)	Sterilizer etc.	7681–52-9	74.44	+^(1)^ -^(12)^	+^(1)^	-^(9)^			
**174**	hypobromous acid water	Sterilizer	13517–11-8	96.91	-^FSC29^	-^FSC29^				5,5-Dimethylhydantoin was used for the assays.
175	sodium hydrosulfite (hydrosulfite)	bleaching agent etc.	7775–14-6	174.11	-^(2)(12)^	-^(2)^				
**176**	2,3-diethylpyrazine	Flavoring agent	15707–24-1	136.19	-^H15^	+^H15^	+^H16^			
**177**	2,3-diethyl-5-methylpyrazine	Flavoring agent	18138–04-0	150.22	-^H17^	-^H17^	-^H18^			
178	allyl cyclohexylpropionate	Flavoring agent	2705-87-5	196.29	-^(15)^	-^H22^				
179	l-cystein monohydrochloride	Antioxidant etc.	7048–04-6	175.64	+^(4)(14)^	+^(4)^	-^(9)^	-^H21^		Target organs for TGR in mice:liver, stomach (glandular stomach)
180	disodium 5′-cytidylate (sodium 5′-cytidylate)	Seasoning	6757–06-8	367.16	-^(1)(19)^	+^(1)^	-^H22^			
181	citral	Flavoring agent	5392–40-5	152.23	-^(3)(4)(14)^	-^(3)(4)^				
182	citronellal	Flavoring agent	106–23-0	154.25	-^(15)^	-^(5)^	-^(8)^			
183	citronellol	Flavoring agent	106–22-9	156.27	-^(15)^	-^H22^				
184	1,8-cineole (eucalyptol)	Flavoring agent	470–82-6	154.25	-^(15)^	-^H22^				
185 [29]	diphenyl (biphenyl)	Antimolding agent	92–52-4	154.21	-^(1)(10)^	-^(1)^				
186	butylated hydroxytoluene	Antioxidant	128–37-0	220.35	-^(10)^	-^(1)^				
187	dibenzoyl thiamine	Dietary supplement	299–88-7	490.58	-^(5)(12)^	-^(5)^				
188	dibenzoyl thiamine hydrochloride	Dietary supplement	35660–60-7	527.04	-^(1)^-^H26^	-^(1)^+^H26^				
**189**	fatty acids	Flavoring agent								
190	aliphatic higher alcohols	Flavoring agent			-^(14)^					
**191**	aliphatic higher aldehydes (except those generally recognized as highly toxic)	Flavoring agent								
**192**	aliphatic higher hydrocarbons (except those generally recognized as highly toxic)	Flavoring agent								
**193**	2,3-dimethylpyrazine	Flavoring agent	5910-89-4	108.14	-^FSC30^	-^H16^				
**194**	2,5-dimethylpyrazine	Flavoring agent	123–32-0	108.14	-^FSC31^		-^H16^			
**195**	2,6-dimethylpyrazine	Flavoring agent	108–50-9	108.14	-^FSC32^		-^H16^			
**196**	2,6-dimethylpyridine	Flavoring agent	108–48-5	107.15	-^FSC33^	-^H18^	-^H18^			
197	oxalic acid	Processing agent	6153-56-6	126.07	-^(3)(13)^	-^(3)^				
198	potassium bromate	Flour treatment agent	7758-01-2	167.00	+^(1)^ -^(12)^	+^(1)^	+^(9)^			
199	*dl*-tartaric acid (*dl* -tartaric acid)	Acidifier	133–37-9	150.09	-^(15)^	-^H23^				
200	*l*-tartaric acid (*d* -tartaric acid)	Acidifier	87–69-4	150.09	-^(2)(15)^	*-* ^(2)^				Identical to *d*-tartaric acid
**201**	potassium *dl*-bitartrate (potassium hydrogen *dl*-tartrate or potassium hydrogen *dl*-tartrate)	Processing agent etc.			-^H22^	-^H22^				
202	potassium *l*-bitartrate (potassium hydrogen l-tartrate or potassium hydrogen *d* -tartrate)	Raising agent	868–14-4	188.18	-^(2)(19)^	*-* ^(2)^				Identical to potassium hydrogen *d*-tartarate
**203**	disodium *dl*-tartrate (disodium *dl*-tartrate)	Processing agent etc.								
204	disodium *l*-tartrate (disodium *l* -tartrate)	Seasoning	6106-24-7	194.05	-^(1)(19)^	+^(1)^	-^(9)^			Identical to disodium *d*-tartarate
205	potassium nitrate	Fermentation regulator etc.	7757-79-1	101.10	-^(4)(13)^	-^(4)^				
206	sodium nitrate	Fermentation regulator etc.	7631-99-4	84.99	-^(1)(13)^	+^(1)^	-^H21^			
207	food red no.2 (amaranth) and its aluminum lake	Food color	915–67-3	604.48	-^(1)(18)^	+^(1)^				
208	food red no.3 (erythrosine) and its aluminum lake	Food color	16423–68-0	897.87	-^(1)(18)^	+^(1)^	*-* ^(2)^			
209	food red no.40 (allura red ac) and its aluminum lake	Food color	25956–17-6	496.42	-^(20)^		-^H20^	-^H20^, -^H23^	Comet: -^H20^, -^H23^	Target organs for Comet in mice:liver, stomach (glandular stomach).Target organs for TGR in mice:liver, stomach (glandular stomach) for (H20), liver, colon for (H23)
210	food red no.102 (new coccine)	Food color	2611–82-7	631.50	-^(1)(18)^	+^(1)^	*-* ^(2)^	-^H20^	Comet: (+)^H20^	Target organs for Comet in mice:liver; pseudo positive, stomach (glandular stomach); negative. Target organs for TGR in mice:liver, stomach (glandular stomach)
211	food red no.104 (phloxine)	Food color	18472–87-2	829.63	-^(1)(18)^	-^(1)^		-^H20^	Comet: +^H20^	Target organs for Comet in mice:liver; pseudo positive, stomach (glandular stomach) in mice; positive. Target organs for TGR in mice:liver, stomach (glandular stomach)
212	food red no.105 (rose bengale)	Food color	632–69-9	1017.64	-^(1)(18)^	-^(1)^		-^H20^	Comet: +^H20^	Target organs for Comet:liver, stomach (glandular stomach) in mice, positive for both. Target organs for TGR in mice:liver, stomach (glandular stomach)
213	food red no.106 (acid red)	Food color	3520-42-1	580.65	-^(1)(18)^	+^(1)^	*-* ^(2)^			
214	food yellow no.4 (tartrazine) and its aluminum lake	Food color	1934-21-0	534.37	-^(1)(18)^	+^(1)^	*-* ^(9)^			
215	food yellow no.5 (sunset yellow fcf) and its aluminum lake	Food color	2783-94-0	452.37	-^(1)(18)^	+^(1)^	-^H21^			
216	food green no.3 (fast green fcf) and its aluminum lake	Food color	2353-45-9	808.85	-^(1)(18)^	-^(1)^	-^(2)^			High purity sample was used in (1).
217	food blue no.1 (brilliant blue fcf) and its aluminum lake	Food color	3844-45-9	792.85	-^(1)(18)^	+^(1)^	-^H21^			
218	food blue no.2 (indigo carmine) and its aluminum lake	Food color	860–22-0	466.35	-^(1)(18)^	+^(1)^				
219	sucrose esters of fatty acids	Emulsifier			-^(1)(20)^	±^(1)^				
220	silicone resin (polydimethylsiloxane)	Antiforming agent			-^(2)^	*-* ^(2)^				
221	cinnamyl alcohol (cinnamic alcohols)	Flavoring agent	104–54-1	134.18	-^(16)^	+^H24^				
222	cinnamaldehyde (cinnamic aldehyde)	Flavoring agent	14371–10-9	132.16	+^(3)^–^(14)^	+^(3)^	-^(9)^	-^H22^		Target organs for TGR:liver, small intestine (Jejunum) in mice
223	potassium hydroxide (caustic potash)	Processing agent	1310-58-3	56.11	-^(17)^					
224	calcium hydroxide (slaked lime)	Processing agent etc.	1305-62-0	74.09	-^(5)(14)^	-^(5)^				
225	sodium hydroxide (caustic soda)	Processing agent	1310-73-2	40.00	-^(16)^					
**226**	magnesium hydroxide	Dietary supplement etc.	1309–42-8	58.32	-^FSC34^					
**227** [30]	sucralose (trichlorogalactosucrose)	Sweetener	56038–13-2	397.64	*-* ^(24)^		*-* ^(24)^			
**228**	calcium stearate	Dietary supplement	1592–23-0	324.56						
**229**	magnesium stearate	Emulsifier etc.	557–04-0	591.24	-^H13^	-^H13^	-^H13^			See calcium stearate
230	calcium stearoyl lactylate (calcium stearyl lactylate)	Emulsifier	5793-94-2		-^(3)^	-^(3)^				
**231**	sodium stearoyl lactylate	Emulsifier etc.	25383–99-7	378.53						See calcium stearoyl lactylate
232	sorbitan esters of fatty acids	Emulsifier			-^(1)(20)^	±^(1)^				
233	d-sorbitol (d-sorbit)	Sweetener etc.	50–70-4	182.17	-^(1)(11)^	-^(1)^				d-Sorbit WP was also used in (1).
234	sorbic acid	Preservative	110–44-1	112.13	-^(2)(16)^	-^(2)^				
235	potassium sorbate	Preservative	24634–61-5	150.22	-^(1)(11)^	+^(1)^	-^H20^			
**236**	calcium sorbate	Preservative etc.	7492-55-9	262.32						See potassium sorbate
237	ammonium carbonate	Processing agent etc.	506–87-6	96.09	-^(19)^	-^H23^				
238	potassium carbonate (anhydrous)	Processing agent etc.	584–08-7	138.21	-^(4)(19)^	-^(4)^				
239	calcium carbonate	Processing agent etc.	471–34-1	100.09	-^(12)^	+^H23^				
240	ammonium bicarbonate (ammonium hydrogen carbonate)	Raising agent etc.	1066-33-7	79.06	-^(4)(19)^	-^(4)^				
241	sodium bicarbonate (bicarbonate soda or sodium hydrogen carbonate)	Raising agent etc.	144–55-8	84.01	-^(2)(19)^	-^(2)^				
242	sodium carbonate (crystal: carbonate soda, anhydrous: soda ash)	Processing agent	497–19-8	105.99	-^(3)(19)^	-^(3)^				Crystalline form was used in (3), and anhydrous form was used for in (19).
243	magnesium carbonate	Processing agent	546–93-0	84.31	-^(5)(13)^	-^(5)^				
244	thiabendazole	Antimolding agent	148–79-8	201.25	-^(1)(10)^	-^(1)^				
245	thiamine hydrochloride (vitamin B1 hydrochloride)	Dietary supplement	67–03-8	337.27	-^(2)(11)^	-^(2)^				
246	thiamine mononitrate (vitamin B1 mononitrate)	Dietary supplement	532–43-4	327.36	-^(5)(15)^	-^(5)^				
247	thiamine dicetylsulfate (vitamin B1 dicetylsulfate)	Dietary supplement			-^(1)^	-^(1)^				
**248**	thiamine thiocyanate (vitamin B1 thiocyanate)	Dietary supplement	130131–60-1	341.45						See thiamine hydrochloride
249	thiamine naphthalene-1,5-disulfonate (vitamin B1naphthalene-1,5-disulfonate)	Dietary supplement			-^(2)^	-^(2)^				
250	thiamine dilaurylsulfate (vitamin B1 dilaurylsulfate)	Dietary supplement etc.			-^(2)^	-^(2)^				
**251**	thioethers (except those generally recognized as highly toxic)	Flavoring agent								
**252**	thiols (thioalcohols) (except those generally recognized as highly toxic)	Flavoring agent								
253	l -theanine	Seasoning	3081-61-6	174.20	-^(4)(19)^	-^(4)^				
254	decanal (decyl aldehyde)	Flavoring agent	112–31-2	156.27	-^(3)(16)^	-^(3)^				
255	decanol (decyl alcohol)	Flavoring agent	112–30-1	158.28	-^(16)^	-^H25^				
256	ethyl decanoate (ethyl caprate)	Flavoring agent	110–38-3	200.32	-^(16)^	-^H25^				
257	sodium iron chlorophyllin	Food color			-^(1)^	-^(1)^				
**258**	5,6,7,8-tetrahydroquinoxaline	Flavoring agent	34413–35-9	134.18	-^H16^	-^H16^				
**259**	2,3,5,6-tetramethylpyrazine	Flavoring agent	1124–11-4	136.20	-^FSC35^					
260	sodium dehydroacetate	Preservative	4418-26-2	208.14	-^(1)(11)^	+^(1)^	+^(9)^			
261	terpineol	Flavoring agent	8000-41-7	154.25	-^(16)^	-^H22^				
**262**	terpene hydrocarbons	Flavoring agent								
263	sodium carboxymethylstarch	Thickening agents			-^(3)^	-^(3)^				
264	copper salts (limited to copper gluconate and cupric	Dietary supplement			-^(13)^					
265	sodium copper chlorophyllin	Food color	28302–36-5	722.13	-^(1)(13)^	-^(1)^				
266	copper chlorophyll	Food color	15739–09-0		-^(1)^	-^(1)^				
267	dl -α-tocopherol	Antioxidant	50–02-9	430.71	-^(1)(10)^	-^(1)^				
**268**	all-racemic-α-tocopheryl acetate	Dietary supplement etc.			-^FSC36^					Identical to *dl*-α-tocopheryl acetate
**269**	*R,R,R* -α-tocopheryl acetate	Dietary supplement etc.								See all-racemic -α-tocopheryl acetate
270	dl-tryptophan	Dietary supplement etc.	54–12-6	204.23	-^(2)(15)^	-^(2)^				
271	l-tryptophan	Dietary supplement etc.	73–22-3	204.23	-^(2)(15)^	-^(2)^				
**272**	trimethylamine	Flavoring agent	75–50-3	59.11	-^H11^	+^H11^	-^H17^			
**273**	2,3,5-trimethylpyrazine	Flavoring agent	14667–55-1	122.17	-^H15^	-^H15^				
274	dl-threonine	Dietary supplement etc.	80–68-2	119.12	-^(2)(15)^	-^(2)^				
275	l -threonine	Dietary supplement etc.	72–19-5	119.12	-^(2)(15)^	-^(2)^				
**276**	nisin	Preservative etc.	1414–45-5	3354.07	-^FSC37^		-^FSC37^			
**277**	natamycin	Preservative	7681-93-8	665.73	-^FSC38^	+^FSC38^				
278	sodium methoxide (sodium methylate)	Processing agent	124–41-4	54.02	-^(14)^					
279	nicotinic acid (niacin)	Dietary supplement etc.	59–67-6	123.11	-^(3)(11)^	-^(3)^	-^(9)^			
280	nicotinamide (niacinamide)	Dietary supplement etc.	98–92-0	122.12	-^(2)(11)^	-^(2)^				
281	sulfur dioxide (sulfurous acid, anhydride)	Preservative etc.	7446-09-5	64.06						
282	chlorine dioxide	Flour treatment agent	10049–04-4	67.45	+^(4)^–^(4)^	+^(4)^	+^(9)^			Ames showed positive for liquid form and negative for powdery form, CA showed numerical abnormality for powdery form, liquid form was used in MN
283	silicon dioxide (silica gel)	Filtration aid	14464–46-1	60.08	-^(20)^					
**284**	carbon dioxide (carbonic acid, gas)	Preservative	124–38-9	44.01						
285	titanium dioxide	Food color	13463–67-7	79.87	-^(19)^	-^H22^				
286	lactic acid	Acidifier	50–21-5	90.08	-^(3)(18)^	-^(3)^				Samples were distinguished whether in a glass container or a plastic container
**287**	potassium lactate	Seasoning etc.	996–31-6	128.17		-^FSC39^			Rec assay: -^FSC39^	
288	calcium lactate	Sweetener etc.	814–80-2	218.22	-^(13)^	-^H23^				
289	iron lactate	Dietary supplement	5905-52-2	233.99	-^(18)^ + ^(5)^	+^(5)^	-^(7)^	-^H23^		Target organs for TGR in mice:liver, kidneys
290	sodium lactate	Acidifier etc.	72–17-3	112.06	-^(4)(19)^	-^(4)^				
**291**	neotame	Sweetener etc.	165450–17-9	378.46	-^FSC40^	-^FSC40^	-^FSC40^			
292	γ-nonalactone (nonalactone)	Flavoring agent	104–61-0		-^(16)^	-^H22^				
293	potassium norbixin	Food color	33261–80-2	456.66	-^(1)^	-^(1)^				
294	sodium norbixin	Food color	33261–81-3	424.45	-^(1)^	-^(1)^				
295	vanillin	Flavoring agent	121–33-5	152.15	-^(2)(16)^	-^(2)^				
296	isobutyl p -hydroxybenzoate	Preservative	4247–02-3	194.23	-^(1)(16)^	-^(1)^				
297	isopropyl p -hydroxybenzoate	Preservative	4191-73-5	180.20	-^(1)(16)^	-^(1)^				
298	ethyl p -hydroxybenzoate	Preservative	120–47-8	166.17	-^(1)(16)^	+^(1)^				
299	butyl p -hydroxybenzoate	Preservative	94–26-8	194.23	-^(1)(10)^	-^(1)^				
300	propyl p -hydroxybenzoate	Preservative	94–13-3	180.20	-^(16)^	+^H25^				
301	p -methylacetophenone	Flavoring agent	122–00-9	134.18	-^(16)^	+^H24^				
302	l -valine	Dietary supplement etc.	72–18-4	117.15	-^(3)(18)^	-^(3)^				
**303**	valeraldehyde	Flavoring agent	110–62-3	86.13	-^FSC41^		-^H17^			
304	calcium pantothenate	Dietary supplement	137–08-6	476.53	-^(3)(12)^	-^(3)^				
305	sodium pantothenate	Dietary supplement	75033–16-8		-^(5)(11)^	-^(5)^				
**306**	biotin	Dietary supplement	58–85-5	244.31	-^FSC42^					identical to *d*-biotin
307	l -histidine monohydrochloride	Dietary supplement	7048–02-4	209.63	-^(5)^	-^(5)^				
**308**	bisbentiamine (benzoylthiamine disulfide)	Dietary supplement	2667-89-2	770.92	-^(4)^ -^H23^	-^(4)^ ± ^H23^				
309	vitamin A (retinol)	Dietary supplement	68–26-8	286.45	-^(20)^	-^H23^				
310	vitamin a fatty acids esters (retinol esters of fatty acids esters)	Dietary supplement			-^(1)^	-^(1)^	-^(9)^			
**311**	1-hydroxyethylidene-1, 1-diphosphonic acid	Processing agent	2809-21-4		-^FSC19^	-^FSC19^				
312	hydroxycitronellal	Flavoring agent	107–75-5	172.26	-^(20)^	+^H24^				
**313** [31]	hydroxycitronellal dimethylacetal	Flavoring agent	141–92-4	218.33	*-* ^(25)^		*-* ^(25)^			
**314**	hydroxypropyl distarch phosphate	Thickening agents etc.	5324-00-8							Evaluated as modified starch
**315**	hydroxypropyl cellulose	Emulsifier etc.	9004–64-2		-^FSC43^					
**316**	hydroxypropyl starch	Thickening agents etc.	68130–14-3							Evaluated as modified starch
**317**	hydroxypropyl methylcellulose	Emulsifier etc.	9004–65-3		-^H12^	-^H12^	-^H12^			
**318**	piperidine	Flavoring agent	110–89-4	85.15	-^FSC44^	-^H17^	-^H17^			
319	piperonal (heliotropine)	Flavoring agent	120–57-0	150.13	+^(16)^		-^H22^	-^H23^		Target organs for TGR in mice:liver, kidneys
320	piperonyl butoxide	Insecticide	51–03-6	338.44	-^(1)(19)^	-^(1)^				
**321**	sunflower lecithin	Emulsifier	8002–43-5		-^FSC45^	-^FSC45^				
322	acetic acid, glacial	Acidifier	64–19-7	60.05	-^(4)(17)^	-^(4)^				
**323**	pyrazine	Flavoring agent	290–37-9	80.09	-^FSC46^	+^FSC46^	-^H18^			
324	pyridoxine hydrochloride (vitamin B6)	Dietary supplement	58–56-0	205.64	-^(2)(11)^	-^(2)^				
**325**	pyrimethanil	Preservative etc.	131341–86-1	199.26	-^FSC47^		-^FSC47^		Rec assay: -^FSC47^	
326	potassium pyrosulfite (potassium hydrogen sulfite or potassium metabisulfite)	Preservative etc.	16731–55-8	222.33	-^(1)(12)^	-^(1)^				
327	sodium pyrosulfite (sodium metabisulfite, acid sulfite of soda)	Preservative etc.	7681–57-4	190.11	-^(3)(19)^	-^(3)^				Described as sodium bisulfite, anhydrous
**328**	pyrrolidine	Flavoring agent	123–75-1	71.12	-^FSC48^	+^H17^	-^H18^			
329	potassium pyrophosphate (tetrapotassium pyrophosphate)	Processing agent	7320–34-5	330.34	-^(15)^	-^H22^				
330	calcium dihydrogen pyrophosphate (acid calcium pyrophosphate)	Dietary supplement etc.	14866–19-4	216.04	-^(20)^	-^H23^				
331	disodium dihydrogen pyrophosphate (acid disodium pyrophosphate)	Processing agent	7758-16-9	221.94	-^(4)(20)^	-^(4)^				
332	ferric pyrophosphate	Dietary supplement etc.	1332-96-3	745.21	-^(4)(11)^	-^(4)^				
333	sodium pyrophosphate (tetrasodium pyrophosphate)	品質改良剤	7722-88-5	265.90	-^(5)(15)^	-^(5)^				
**334**	pyrrole	Flavoring agent	109–97-7	67.09	-^FSC49^	+^H18^	-^H21^			
335	l -phenylalanine	Dietary supplement etc.	63–91-2	165.19	-^(2)(18)^	-^(2)^				
336	isoamyl phenylacetate	Flavoring agent	102–19-2	206.28	-^(16)^	-^H25^				
337	isobutyl phenylacetate	Flavoring agent	102–13-6	192.25	-^(19)^	-^H24^				
338	ethyl phenylacetate	Flavoring agent	101–97-3	164.20	-^(2)(16)^	-^(2)^				
**339**	2-(3-phenylpropyl) pyridine	Flavoring agent	2110-18-1	197.28	-^H17^	+^H17^	-^H18^			
**340**	phenethylamine	Flavoring agent	64–04-0	121.18	-^H17^	+^H17^	-^H18^			
**341**	phenol ethers (except those generally recognized as highly toxic)	Flavoring agent								
**342**	phenols (except those generally recognized as highly toxic)	Flavoring agent								
**343**	ferrocyanides (potassium ferrocyanide (potassium hexacyanoferrate (ii)), calcium ferrocyanide (calcium hexacyanoferrate (ii)), sodium ferrocyanide (sodium hexacyanoferrate (ii)))	Processing agent etc.	13943–58-3, 13821–08-4, 13601–19-9	422.39, 508.29, 484.06	*–*	*–*				Evaluated as potassium ferrocyanide and sodium ferrocyanide
**344**	butanol	Processing agent etc.	71一36–3	74.12	-^H15^	-^H15^	-^H15^			
**345**	butylamine	Flavoring agent	109–73-9	73.14	-^FSC50^	+^H17^	-^H18^			
**346**	butyraldehyde	Flavoring agent	123–78-8	72.11	-^FSC51^	+^FSC51^ -^FSC51^	-^FSC51^			
347	butylated hydroxyanisole	Antioxidant	25013–16-5	180.24	-^(1)(10)^	-^(1)^	-^(3)^			
348	fumaric acid	Acidifier	110–17-8	116.07	-^(4)(17)^	-^(4)^				
349	monosodium fumarate (sodium fumarate)	Acidifier	5873-57-4	138.05	-^(5)(19)^	-^(5)^				
**350**	fludioxonil	Preservative etc.	131341–86-1	248.19	-^FSC52^	+^FSC52^	-^FSC52^			
351	furfurals and its derivatives (except those generally recognized as highly toxic)	Flavoring agent			-^(17)^	-^H23^				Furfural was used for the assay.
**352**	propanol	Processing agent etc.	71–23-8	60.09	-^FSC53^					
**353**	propionaldehyde	Flavoring agent	123–38-6	58.08	-^FSC54^		-^H17^			
354	propionic acid	Flavoring agent etc.	79–09-4	74.08	-^(16)^	-^H22^				
355	isoamyl propionate	Flavoring agent	105–68-0	144.21	-^(3)(16)^	-^(3)^				
356	ethyl propionate	Flavoring agent	105–37-3	102.13	-^(3)(16)^	-^(3)^				
357	calcium propionate	Preservative	4075-81-4	186.22	-^(2)(16)^	-^(2)^				
358	sodium propionate	Preservative	137–40-6	96.06	-^(1)(11)^	-^(1)^				
359	benzyl propionate	Flavoring agent	122–63-4	164.20	-^(16)^	-^H24^				
360	propylene glycol	Quality sustainer etc.	57–55-6	76.09	-^(1)(14)^	+^(1)^	-^(9)^			
361	propylene glycol esters of fatty acids	Emulsifier			-^(2)(20)^	-^(2)^				
362	hexanoic acid (caproic acid)	Flavoring agent	142–62-1	116.16	-^(12)^	-^H24^				
363	allyl hexanoate (allyl caproate)	Flavoring agent	123–68-2	156.22	-^(17)^	-^(4)^ -^H22^				
364	ethyl hexanoate (ethyl caproate)	Flavoring agent	123–66-0	144.21	-^(17)^	-^(4)^ -^H24^				
365	ethyl heptanoate (ethyl enanthate)	Flavoring agent	106–30-9	158.24	-^(12)^	-^H24^				
366	l -perillaldehyde	Flavoring agent	18031–40-8	150.22	-^(3)(19)^	+^(3)^	-^(9)^			
367	benzyl alcohol	Flavoring agent	100–51-6	108.14	-^(3)(17)^	-^(3)^	-^(9)^			
368	benzaldehyde	Flavoring agent	100–52-7	106.12	-^(17)^	-^H22^				
**369**	2-pentanol (sec-amylalcohol)	Flavoring agent	6032-29-7	88.15	-^H16^	-^H16^				
**370**	trans-2-pentenal	Flavoring agent	1576-87-0	84.12			-^H17^			
**371**	1-penten-3-ol	Flavoring agent	616–25-1	86.13	+^H16^	-^H16^	-^H17^			Ames showed positive only in T1537, whose maximum number of the revertants was s within the range of negative control.
**372**	aromatic alcohols	Flavoring agent								
**373**	aromatic aldehydes (except those generally recognized as highly toxic)	Flavoring agent								
374	propyl gallate	Antioxidant	121–79-9	212.20	-^(1)^ + ^(13)^	+^(1)^	-^H21^	-^H21^		Target organs for TGR in mice:liver, stomach (glandular stomach)
375	sodium polyacrylate	Thickening agents etc.	9003–04-7		-^(2)(13)^	-^(2)^				
376	polyisobutylene (butyl rubber)	Chewing gum base	9003–27-4		-^(3)^	-^(3)^				
**377**	polysorbate 20	Emulsifier etc.	9005-64-5	1227.72						See the data on substances with different molecular weight
**378**	polysorbate 60	Emulsifier etc.	9005-67-8	1311.90	-^FSC55^	-^FSC55^				
**379**	polysorbate 65	Emulsifier etc.	9005-71-4	1842	-^FSC55^	+^FSC55^	-^FSC55^			
**380**	polysorbate 80	Emulsifier etc.	9005-65-6	1309.68	-^FSC55^	-^FSC55^	-^FSC55^		Rec assay: -^FSC55^	
**381**	polyvinylpyrroridone	Thickening agents etc.	9003-39-8		-^FSC56^					
**382**	polyvinylpolypyrrolidone	Processing agent	25249–54-1		-^FSC57^					
383	polybutene (polybutylene)	Chewing gum base	9003–28-5		-^(4)(20)^	-^(4)^				
384	potassium polyphosphate	Processing agent	68956–75-2		-^(20)^	-^H22^				
385	sodium polyphosphate	Processing agent	68915–31-1		-^(3)(5)(15)^	-^(3)(5)^				Sodium tripolyphosphate was used in (3).
386	d -borneol	Flavoring agent	464–43-7	154.25	-^(17)^	-^H22^				
387	maltol	Flavoring agent	118–71-8	126.11	-^(4)^ + ^(17)^	+^(4)^	+^(9)^	-^H21^		Target organs for TGR in mice:liver, stomach (glandular stomach)
388	d-mannitol (d-mannite)	Antisticking agent etc.	69–65-8	182.17	-^(3)(13)^	-^(3)^				
389	potassium metaphosphate	Processing agent	7790–53-6	118.07	-^(2)(15)^	-^(2)^				
390	sodium metaphosphate	Processing agent	10361–03-2	101.96	-^(3)(15)^	-^(3)^				
391	dl-methionine	Dietary supplement etc.	59–51-8	149.21	-^(18)^	-^H22^				
392	l-methionine	Dietary supplement	63–68-3	149.21	-^(18)^	-^H23^				
393	methyl n-methylanthranilate	Flavoring agent	85–91-6	165.19	-^(17)^	-^H22^				
**394**	5-methylquinoxaline	Flavoring agent	13708–12-8	144.17	-^H15^	+^H15^	-^H15^			
**395**	6-methylquinoline	Flavoring agent	91–62-3	143.19			-^H16^			
**396**	6,7-dihydro-5-methyl-5 h-cyclopentapyrazine	Flavoring agent	23747–48-0	134.18	-^H16^	-^H16^	-^H17^			
397	methyl cellulose	Thickening agents etc.	9004–67-5		-^(3)(5)(14)^	-^(3)^				
**398**	1-methylnaphthalene	Flavoring agent	90–12-0	142.20	-^FSC58^	+^H17^	-^H18^	-^FSC58^		Target organs for TGR in mice:lung
399	methyl β-naphthyl ketone	Flavoring agent	93–08-3	170.21	-^(17)^	-^H24^				
**400**	2-methypyrazine	Flavoring agent	109–08-0	94.11	-^FSC59^		-^H16^			
**401**	2-methylbutanol	Flavoring agent	137–32-6	88.15	-^H15^	-^H15^	-^H15^			
**402**	3-methyl-2-butanol	Flavoring agent	598–75-4	88.15	-^H17^	-^H17^				
**403**	2-methylbutyraldehyde	Flavoring agent	96–17-3	86.13	-^FSC60^		-^H17^			
**404**	trans −2-methyl-2-butenal, (e)-2-methyl-2-butenal	Flavoring agent	497–03-0	84.12	+^H17^	+^H17^	-^H18^			
**405**	3-methyl-2-butenal	Flavoring agent	107–86-8	84.12	+^H16^	+^H16^	-^H17^			
**406**	3-methyl-2-butenol	Flavoring agent	556–82-1	86.13	+^H16^	+^H16^	+^H17^			The result of MN was pseudo positive.
407	methyl hesperidin (soluble vitamin P)	Dietary supplement	11013–97-1	624.59	-^(3)(12)^	-^(3)^				
408	dl-menthol (dl-peppermint camphor)	Flavoring agent	89–78-1	156.27	-^(1)(20)^	-^(1)^				
409	l-menthol (peppermint camphor)	Flavoring agent	2216–51-5	156.269	-^(4)(17)^	-^(4)^				
410	morpholine salts of fatty acids	Coating agent			-^(2)^	-^(2)^				
411	folic acid	Dietary supplement	59–30-3	441.404	-^(5)(11)^	-^(5)^				
412	butyric acid	Flavoring agent	107–92-6	88.106	-^(3)(17)^	-^(3)^				
413	isoamyl butyrate	Flavoring agent	106–27-4	158.241	-^(3)(4)(17)^	-^(3)(4)^				
414	ethyl butyrate	Flavoring agent	105–54-4	116.16	-^(3)(4)(17)^	-^(3)(4)^				
415	cyclohexyl butyrate	Flavoring agent	1551-44-6	170.252	-^(17)^	-^H25^				
416	butyl butyrate	Flavoring agent	109–21-7	144.214	-^(17)^	-^H24^				
**417**	lactones (except those generally recognized as highly toxic)	Flavoring agent								
418	l-lysine l-aspartate	Dietary supplement etc.			-^(5)^	-^(5)^				
419	l-lysine monohydrochloride	Dietary supplement etc.	657–27-2	182.65	-^(5)(18)^	-^(5)^				
420	l-lysine l-glutamate	Dietary supplement etc.			-^H23^	-^H23^				
421	linalool	Flavoring agent	78–70-6	154.25	-^(3)(17)^	-^(3)^				
**422**	calcium 5′-ribonucleotide	Seasoning			-^H22^	-^H22^				
423	disodium 5′-ribonucleotide (sodium 5′-ribonucleotide)	Seasoning			-^(3)^	-^(3)^				
424	riboflavin (vitamin B2)	Dietary supplement etc.	83–88-5	376.369	-^(1)(6)(11)^	+^(1)^	-^(9)^		Rec assay: -^(6)^	
425	riboflavin tetrabutyrate (vitamin B2 tetrabutyrate)	Dietary supplement etc.	752–56-7	656.733	-^(4)(18)^	-^(4)^				
426	riboflavin 5′-phosphate sodium (riboflavin phosphate sodium, vitamin B2 phosphate sodium)	Dietary supplement etc.	130–40-5	478.33	-^(3)(18)^+^(5)^	-^(3)^	-^(7)^			
427	sulfuric acid	Processing agent	7664-93-9	98.072	-^(16)^					
428	aluminum ammonium sulfate (crystal: ammonium alum, desiccated: burnt ammonium alum)	Raising agent etc.	7784–25-0	237.15	-^(14)^	-^H20^	-^H20^			
429	aluminum potassium sulfate (crystal: alum or potassium alum, desiccated: burnt alum)	Raising agent etc.	10043–67-1	258.21	-^(3)(14)^	-^(3)^	-^H20^			
430	ammonium sulfate	Processing agent	7783-20-2	132.14	-^(18)^	-^H23^				
**431**	potassium sulfate	Seasoning etc.	7778–80-5	174.25						See substances with different salt
432	calcium sulfate	Tofu coagulator etc.	7778–18-9	172.17	-^(13)^	-^H23^				
433	ferrous sulfate	Dietary supplement etc.	13463–43-9	151.91	-^(3)(19)^	+^(3)^	-^(9)^			
434	sodium sulfate	Processing agent	7757-82-6	142.036	-^(18)^	-^H23^				
435	magnesium sulfate	Tofu coagulator etc.	7487-88-9	120.361	-^(3)(18)^	-^(3)^				Dried sample was also used in (3)
436	dl-malic acid (dl -malic acid)	Acidifier	6915-15-7	134.087	-^(2)(17)^	-^(2)^				
437	sodium dl-malate (sodium dl -malate)	Acidifier etc.	138–09-0	178.051	-^(4)(19)^	-^(4)^				
438	phosphoric acid	Acidifier	7664-38-2	97.994	-^(18)^	-^H23^				
**439**	distarch phosphate	Thickening agents etc.	–		-^FSC20^	-^FSC20^	-^FSC20^			
**440**	monostarch phosphate	Thickening agents etc.	–		-^FSC20^	-^FSC20^	-^FSC20^			
441	tripotassium phosphate (potassium phosphate, tribasic)	Processing agent	7778-53-2	212.264	-^(19)^					
442	tricalcium phosphate (calcium phosphate, tribasic)	Processing agent etc.	7758-87-4	310.18	-^(13)^					
**443**	trimagnesium phosphate	Processing agent	10233–87-1	262.86						See substances with different salt
444	diammonium hydrogen phosphate (diammnonium phosphate or ammnonium phosphate, dibasic)	Processing agent	7783-28-0	132.056	-^(18)^	-^H23^				
445	ammonium dihydrogen phosphate (ammonium phosphate,monobasic or monoammonium phosphate)	Processing agent	7722-76-1	115.025	-^(5)(18)^	+^(5)^	-^(7)^			
446	dipotassium hydrogen phosphate (dipotassium phosphate or potassium phosphate, dibasic)	Processing agent	7758-11-4	174.174	-^(19)^					
447	potassium dihydrogen phosphate (monopotassium	Processing agent	7778–77-0	136.084	-^(18)^	-^H22^				
448	calcium monohydrogen phosphate (calcium phosphate,	Processing agent	136.06	136.06	-^(5)(12)^	-^(5)^				
449	calcium dihydrogen phosphate (calcium phosphate,	Processing agent	7758-23-8	234.05	-^(3)(14)^	-^(3)^				
450	disodium hydrogen phosphate (disodium phosphate)	Processing agent	7558-79-4	141.958	-^(3)(19)^	-^(3)^				Crystal form and anhydrous form were used in (3) and in (19), respectively.
451	sodium dihydrogen phosphate (monosodium phosphate)	Processing agent	7558-80-7	119.976	-^(18)^					
**452**	magnesium monohydrogen phosphate	Processing agent etc.	7782–75-4	174.33						See compounds with different salt
453	trisodium phosphate (sodium phosphate, tribasic)	Processing agent	7601-54-9	163.94	-^(19)^					Anhydrous substrate was also used in the assay
**454**	phosphated distarch phosphate	Thickening agents etc.	–							See substrate with different salt

^a^Numbers are consistent with those shown in Tables 1 and 2

^b^Underlined bold style means that the items are added as indicated in Table 2

^c^(H12) means Heisei era 12, and indicates that it referred to the report on food inspection expenses implemented in 2000 (=Heisei 12). The same applies to H13, H16, H17, H18, H20, H21, H22, H23, H24, H25, H26

^d^(FSC) indicates Risk assessment reports in Food Safety Commission of Japan. See http://www.fsc.go.jp/fsciis/evaluationDocument/list?itemCategory=000 (Additional file 1)

## How the data were summarized

The following set of three tests has traditionally been used to evaluate mutagenicity of food additives: reverse mutation assay (Ames test) using bacteria; chromosomal aberration test using cell culture (CA); and micronucleus test using mice (MN). The Hayashi report summarized the data from the results of these three tests. Two new tests suitable for the evaluation of food additives were subsequently added in the OECD Genotoxicity Test Guidelines. The two adopted test guidelines are: “Genetic mutation test using transgenic rodent somatic and germ cells (TG 488)” (the TGR test); and “In vivo mammalian alkaline comet assay (TG 489)” (the comet test). Results using these assays are included in this paper (Table 3). While both tests have advantages for the evaluation of genotoxicity in specific tissues, the TGR test is intended as a mutagenicity test (similar to the Ames test) therefore the weighting of TGR results is generally higher due to its high correlation with carcinogenicity.

When searching for items that had not been subjected to mutagenicity testing, it was felt desirable that all test outcomes were discovered without exception. Thus, in Table 3, data from journals other than Hayashi report are included; for example, data published in the Annual Report of the Tokyo Metropolitan Research Laboratory of Public Health (originally published in Japanese). Reports that were surveyed, including journals other than Hayashi report, are listed at the end of this paper. Results of the three tests used initially are mainly copied from the Hayashi report (also published in Japanese). Data reevaluated or added after the Hayashi report include results from outsourced testing laboratories accredited by the MHLW as part of the “Projects for safety of food additives.”

In the main Table, the superscript symbols “^H22^” and “^H23^” indicate results commissioned in tests conducted in Fiscal Year 2010 (FY2010) and FY2011, respectively. Test results from the Risk Assessment Reports prepared by the Food Safety Commission (FSC) are also included; these are indicated by superscript “^FSC^”, and the URL for the Risk Assessment Reports of the item is given in the reference list. Note that reference numbers in the text are not given in numeric order but in the order of appearance in Table 3. The eliminated and newly added substances are listed in Tables 1 and 2, respectively. In Table 3, the item numbers from Table 2 are shown underlined.

## Commentary for 13 items newly subjected to the TGR test

### 1) Five items positive for Ames and chromosome aberration tests while negative for in vivo micronucleus test

#### Sodium nitrite (No. 6 in Table 3)

Ames testing was performed at the highest dose of 10 mg/plate using TA1535, TA98, TA1537, TA94, TA92 in FY1979, and positive results were obtained with TA100 and TA1535 regardless of S9mix [2]. Subsequently, using TA97 and TA102, a statistically significant increase in the number of revertants (maximum dose 10 mg/plate) was reported in both strains regardless of S9mix [3]. However, the result is given as negative in Table 3 since the number of reverted colonies did not reach twice of the number for the negative control. Many positive results of Ames tests were reported in this item, thus, mutagenicity was suspected for this substance [4].

For chromosomal abnormalities, the chromosome aberration test using Chinese Hamster lung (CHL) was performed at a maximum dose of 1.0 mg/mL without S9mix, and strong induction of structural abnormalities was reported [2]. Subsequently, in vivo bone marrow micronucleus tests using ddY mice were carried out under three conditions: a single intraperitoneal (i.p.) dose of 200 mg/kg body weight; four i.p. doses at 50 mg/kg body weight at 24-h intervals; and a single oral dose of 400 mg/kg body weight [5], all results being reported as negative.

In FY2009, TGR testing using *gpt* delta mice was performed in the liver and glandular stomach for confirmation of in vivo mutagenicity. These organs were selected because the liver metabolizes many substances and is highly sensitive in this assay, and the glandular stomach is the organ first exposed to the substance under test with oral administration. The mutagenicity that was previously observed in the Ames test did not occur in vivo, since the TGR results were negative in both organs after 28 days of administration via drinking water at a maximum dose of 5000 mg/kg body weight [H21(FY2009)].

#### l-Cysteine monohydrochloride (No. 179 in Table 3)

Ames testing was carried out using TA100, TA98, TA2637, TA94, at a maximum dose of 10 mg/plate with or without S9mix in FY1982. Positive results were reported for TA100 with S9mix, and for TA2637 with and without S9mix [6]. Chromosomal aberration tests were performed using CHL cells without S9mix and structural abnormalities were induced (maximum dose was 2 mg/mL) [6]. Subsequently, the Ames test was conducted at Tokyo Metropolitan Research Laboratories of Public Health (TMRL) using TA97 and TA102 with and without S9mix at a maximum dose of 10 mg/plate, and positive results were reported under all conditions [7]. Since genotoxicity was detected in vitro, bone marrow micronucleus test using ddY mice was conducted in 1986. The test results were negative for single i.p. doses of 125, 250 and 500 mg/kg body weight [5].

In FY2009, TGR testing using *gpt* delta mice was performed in the liver and glandular stomach for confirmation of in vivo mutagenicity; the results were negative in both organs following oral gavage for 28 days at a maximum dose of 1000 mg/kg body weight [H22(FY2010)].

Despite positive in vitro results, it was concluded that l-cysteine hydrochloride is not genotoxic in living organisms because results were negative in in vivo MN and TGR tests.

#### Cinnamaldehyde (No. 222 in Table 3)

Ames tests were performed at a maximum dose of 0.5 mg/plate with and without S9mix using TA100, TA1535, TA98, TA1537, TA92, TA97 in FY1981. Only TA100 showed positive results regardless of the metabolic activation in this report [8]. In the chromosome aberration test simultaneously carried out using CHL cells, structural abnormalities were induced without S9mix (maximum dose 0.015 mg/mL) [8]. Subsequently, Ames tests were carried out at TMRL using TA97 and TA102 at a maximum dose of 0.1 mg/plate; results were negative regardless of metabolic activation [7]. Since genotoxicity was detected in vitro, in vivo bone marrow MN testing was conducted in FY1986. Mice (ddY) were given a single i.p. injection at 125, 250 and 500 mg/kg body weight and the results were negative [5].

We conducted TGR tests in the liver and small intestine (jejunum) using *gpt* delta mice to confirm in vivo mutagenicity in FY2010 and FY2011. The reason for choosing the small intestine as the target organ is that it is the first in the gastrointestinal tract to be exposed to substances administered orally. Mice were dosed by oral gavage at 125, 250, 500 and 1000 mg/kg body weight for 28 days, and mutagenicity was investigated for the animals dosed at 500 and 1000 mg/kg body weight. Negative results were obtained in both organs [H22(FY2010)].

Despite showing genotoxicity in vitro, it was concluded that cinnamaldehyde does not show genotoxicity in living organisms because results were negative in in vivo MN and TGR tests.

#### Iron lactate (No. 289 in Table 3)

Ames testing was carried out in FY1983 and the results were positive without S9mix in TA97, TA102 and TA2637 at the highest dose of 5.0 mg/plate, and negative in TA100 and TA98 with and without S9mix [9]. Chromosomal aberration testing using CHL cells conducted in the same year induced structural abnormalities without S9mix (maximum dose 2.5 mg/mL) [9]. Subsequently, Ames tests were conducted at TMRL using TA97 and TA102, yielding negative results with and without S9mix [10]. The maximum dose in this study, 1.0 mg/plate, is considered to be insufficient. In vivo MN testing using ddY mice was conducted in FY1986 (single i.p. administration of 30 mg/kg body weight, and four separate i.p. doses of 7.5 mg/kg body weight/day), with negative results [11].

In FY2011, TGR tests in the liver and kidney were carried out using *gpt* delta mice to confirm in vivo mutagenicity. The reason for using the kidney as the target organ is that nephrotoxicity was observed in macroscopic examinations. After doses of 250, 500 and 1000 mg/kg body weight for 28 days by oral gavage, mutation was investigated at doses of 500 and 1000 mg/kg body weight. Since the results were negative in both organs [H23(FY2011)], it was concluded that iron lactate does not induce mutation in vivo.

#### Propyl gallate (No. 374 in Table 3)

Ames testing was carried out in TA100, TA98, TA1537 at 500 μg/plate in FY1979, and negative results were obtained regardless of metabolic activation [2]. Subsequently, Ames tests were carried out at TMRL using TA97 and TA102, and at the maximum dose of 0.1 mg/plate it was found that TA102 showed a statistically significant increase in the number of revertants regardless of metabolic activation [12]. TA100 and TA1535 mainly detect base substitution occurring in GC base pairs, while TA102 mainly reveals substitution in AT base pairs. Thus, these results (negative in TA100 and TA1535, positive in TA102) suggest that propyl gallate is reactive with AT base pairs. The negative results for TA98, TA1537 and TA97 indicate that the probability of inducing a frameshift mutation is low. Five out of six tests showed positive results at doses higher than 50 μg/mL, but an unusual pattern was shown regarding dose correlation for which the mechanism is unknown.

Since the above results suggested that propyl gallate induces base substitution with AT base pairs in vitro, TGR testing was performed in liver and glandular stomach using *gpt* delta mice in FY2009 [H21(FY2009)]. Repeated administration over 28 days produced negative results for both organs at the highest dose of 1000 mg/kg body weight. Thus, the mutagenicity of propyl gallate was detected in vitro, but not considered to be detected in vivo.

After chromosomal aberration testing in FY1979 it was reported that structural abnormalities were induced in CHL after 24 h treatment at a dose of 0.04 mg/mL without S9mix [2]. To investigate the risk of chromosomal aberration, in vivo bone marrow MN testing was conducted in FY2009, with negative results at the maximum dose of 1000 mg/kg body weight (administered twice) [H21(FY2009)]. Therefore, although chromosomal abnormalities were detected in vitro, they were not in vivo.

From the results detailed above, propyl gallate was considered to be non-genotoxic to living bodies.

### 2) Two items negative for chromosome aberration and in vivo micronucleus tests while positive for Ames test

#### Erythorbic acid (isoascorbic acid)(No. 78 in Table 3)

This substance was positive only in TA100 regardless of S9mix (highest dose 50 mg/plate) in the Ames test using the strains TA100, TA98, TA1535, TA98, TA1537, TA92 and TA94 in FY1980 [13]. In chromosomal aberration tests using CHL cells, a negative result was reported at the highest dose of 0.25 mg/mL without S9mix [13]. In the Ames test conducted at TMRL using TA97 and TA102, a statistically significant increase in the number of revertants was reported in both strains regardless of S9mix (maximum dose 10 mg/plate) [14]. However, the result is given as negative in Table 3 since the number of revertants did not reached twice of the number of the negative control. Subsequently, an in vivo bone marrow MN test using ddY mice was performed, and this substance showed negative when administered in a single dose of 1500 mg/kg body weight (at the maximum) or as four treatments (at 24-h intervals) at 750 mg/kg body weight (at the maximum).

Thereafter, TGR testing using *gpt* delta mice was conducted for liver and glandular stomach (maximum dose 1000 mg/kg body weight for 28 days by gavage) in FY2009 in order to investigate in vivo mutagenicity. Neither point mutation nor deletion mutation was induced in either organ [H21(FY2009)]. It was concluded that there are no concerns for genotoxicity of erythorbic acid to living bodies.

#### Piperonal (No. 319 in Table 3)

In Ames testing at TMRL using TA97 and TA102 this substance showed positive results in TA97 without S9mix at the highest dose of 1 mg/plate [3]. It is reported that a statistically significant increase was observed with S9mix, but the level did not reach twice that of the negative control. There are no reports on chromosomal aberration tests. MN testing using ICR mice was carried out in FY2010, and the results were negative in bone marrow after oral administration of 250, 500 and 1000 mg/kg body weight (two doses at 24-h intervals) [H22(FY2010)]. In FY2010–11, TGR testing using *gpt* delta mice was performed in the liver and kidney in order to confirm in vivo mutagenicity at doses of 250, 500 and 1000 mg/kg body weight for 28 days by gavage. The results were negative for both organs at doses of 500 and 1000 mg/kg body weight [H23(FY2011)].

From the above results it was concluded that piperonal does not show genotoxicity in living organisms.

### 3) One item positive in all three tests (Ames, chromosomal aberration and in vivo micronucleus tests)

#### Maltol

In 1982, Ames testing using TA100, TA98, TA2637, and TA94 was carried out for maltol at a maximum dose of 10.0 mg/plate, and the results were negative both with and without S9mix [6]. Chromosomal aberration testing was conducted in the same year, and it was reported that structural abnormalities were induced in CHL cells at the highest dose (0.075 mg/mL) without S9mix [6]. Subsequently, Ames testing was performed at TMRL with TA97 and TA102, at the highest dose of 10.0 mg/plate with and without S9mix. Induction of colony formation at a reversion level almost double that of the negative control was observed in TA97 at a dose of 1 mg/plate without S9mix. Positive judgment has been reported in a micronucleus test using bone marrow of ddY mice, 24 h after single i.p. administration of 125, 250 and 500 mg/kg body weight [5]. Since the usage of this item is limited to fragrances, there is no possibility of exposure in vivo at a concentration equivalent to the dose at which chromosomal abnormality was detected in vitro.

In FY2009, TGR testing using *gpt* delta mice was performed in the liver and glandular stomach for confirmation of in vivo gene mutagenicity. The results were negative in both organs at doses of 400, 200, 100 and 50 mg/kg body weight for 28 days by gavage [H21(FY2009)].

From the above, it seems that there is no concern of genotoxicity in maltol for living bodies.

### 4) Five items for which the Ames test was negative

#### 1-Methylnaphthalene

In FY2005 this substance was reported to have induced structural abnormalities in a chromosome aberration test using CHL cells [H17(FY2005)] while in vivo bone marrow micronucleus testing conducted in FY2006 reported negative results in a two-dose study of 1000 mg/kg body weight at the maximum [H18(FY2006)].

Regarding mutagenicity, in Ames tests using several strains of *Salmonella typhimurium* conducted from 1980 to 2002, all results were negative, whereas a weak positive result was reported in the forward mutation test using *S. typhimurium* (maximum dose 0.992 mg/mL, 2-h exposure) [FSC58]. In theory, the Ames test, which is a reverse mutation test, can detect only specific point mutations while a forward mutation test can detect mutations of any type. Thus, it would be problematic for the negative results of the Ames tests to be taken as completely eliminating the concerns about mutagenicity arising from the result of the forward mutation tests. Subsequently, a TGR test in *gpt* delta mice (males and females) was performed on the lungs. The reason that the lungs were selected as the target organ was that weak carcinogenicity was observed in the lungs of mice in the 81-week chronic toxicity–carcinogenicity combination test reported in 1993. TGR tests were conducted at doses of 170 and 280 mg/kg body weight for females and 120 and 220 mg/kg body weight for males by dietary administration for 13 weeks, the results being negative in all conditions ([15], FSC58).

From the above, 1-methylnaphthalene is considered to have no concerns of genotoxicity for living bodies.

#### Food Red No. 40

In FY1995 at TMRL negative results (maximum dose 10 mg/plate) in Ames tests with TA97 and TA102 with and without S9mix were reported [16]. Chromosomal aberration tests have not been carried out. Subsequently, in vivo micronucleus tests using CD1 mice was performed in FY2008, and results of single oral gavage of 500, 1000 and 2000 mg/kg body weight were reported to be negative in bone marrow [H20(FY2008)].

In FY2008 and FY2011, comet and TGR tests using mice were conducted to examine in vivo DNA damage inducibility and mutagenicity, respectively. In the comet test, CDF_1_ mice were administered two doses of 500, 1000 and 2000 mg/kg body weight by oral gavage with a 24-h interval. The results were negative for both liver and glandular stomach (H20(FY2008), [17]). In addition, another comet assay using ICR mice was carried out with two oral gavage administrations (24-h interval) at doses of 500, 1000 and 2000 mg/kg body weight. The results were negative in both stomach and colon, while an increase without dose correlation was observed in liver [H23(FY2011)]. TGR testing was conducted using the Muta™Mouse, orally gavaged at doses of 250, 500 and 1000 mg/kg body weight for 28 days; mutagenicity in the liver and glandular stomach was not observed [H20(FY2008)]. Furthermore, TGR tests using *gpt* delta mice were carried out and the results were negative for mutagenicity in the large intestine following oral gavage for 28 days at doses of 250, 500 and 1000 mg/kg body weight [H23(FY2011)].

From the above, it seems that there is no concern of genotoxicity of Food Red No. 40 for living bodies.

#### Food Red No. 102

In 1979 negative results were reported following Ames tests carried out with and without S9mix conditions using TA100, TA1535, TA98, TA1537, TA92 and TA94 (maximum dose 5.0 mg/plate) [2]. In chromosomal aberration tests using CHL cells carried out in the same year, induction of structural abnormalities was observed with S9mix (maximum dose of 4.0 mg/mL) [2]. Subsequently, Ames tests were conducted at TMRL using TA97 and TA102, and the results were negative with and without S9mix (maximum dose 10 mg/plate) [10]. Since chromosomal abnormalities were induced in vitro, micronucleus tests using ddY mice were carried out in FY1980. Results were negative for two sets of conditions in bone marrow: single i.p. administration of 300, 600 and 1200 mg/kg body weight; and four i.p. doses of 300 mg/kg body weight [13].

In FY2008, comet and TGR tests using mice were carried out to examine in vivo DNA damage inducibility and mutagenicity, respectively [H20(FY2008)]. Comet tests were carried out by oral gavage (twice, at 24-h intervals) at doses of 500, 1000 and 2000 mg/kg body weight using CDF_1_ mice, and the results were judged as negative in both liver and glandular stomach. The TGR tests were carried out in Muta™Mouse using oral gavage at 250, 500 and 1000 mg/kg body weight for 28 days, and the results were negative in both liver and glandular stomach.

From the above, Food Red No.102 is considered not to have concerns of genotoxicity to living bodies.

#### Food Red No. 104

In 1979, negative results in Ames tests carried out using TA100, TA1535, TA98, TA1537, TA92 and TA94 with and without S9mix (maximum dose 5 mg/plate) were reported [2]. In the same year, chromosomal aberration tests using CHL cells were conducted without S9mix, and the results were negative (maximum dose 0.25 mg/mL) [2]. Ames tests were also conducted at TMRL with TA97 and TA102, and the results were negative with and without S9mix (maximum dose 1 mg/plate) [10]. Micronucleus testing using mice was not performed because both in vitro tests were negative.

In FY2008, comet and TGR tests using mice were carried out to confirm in vivo DNA damage inducibility and mutagenicity, respectively. Comet tests for liver and glandular stomach were performed by oral gavage (twice, 24-h interval) at doses of 250, 500 and 1000 mg/kg body weight on CDF_1_ mice. Results showed false positive in the liver and positive in glandular stomach [H20(FY2008)]. TGR tests were conducted using Muta™Mouse with oral gavage at 250, 500 and 1000 mg/kg body weight for 28 days and liver and glandular stomach were examined for mutation induction; the results were negative in both [H20(FY2008)].

Based on the above results, it is likely that the DNA damage detected in the comet tests would not reach the level necessary to produce mutation. The negative results in liver and glandular stomach in TGR tests support this view, and it seems likely that the DNA damage is repaired in mouse body. Therefore, Food Red No. 104 is considered not to induce genotoxicity (mutagenicity) in vivo.

#### Food Red No. 105

In FY1978 results were negative in Ames tests (maximum dose 5.0 mg/plate) with and without S9mix using TA100, TA1535, TA98, TA1537, TA92 and TA94 [2]. In the same year, chromosomal aberration tests using CHL cells were also carried out (S9mix only) and the results were negative (maximum dose 0.25 mg/mL) [2]. Subsequently, Ames tests were carried out at TMRL using TA97 and TA102 (maximum dose 1 mg/plate) with and without S9mix, with negative results [10]. Micronucleus tests using mice were not carried out because both in vitro tests were negative.

In FY2008, in order to examine in vivo DNA damage inducibility and mutagenicity, comet and TGR tests were conducted, respectively, in mice. The comet test was positive in both liver and glandular stomach for oral administration (twice, 24-h interval) at doses of 250, 500 and 1000 mg/kg body weight for CDF_1_ mice, and were examined [H20(FY2008)]. The TGR test was conducted using Muta™Mouse with oral gavage at 250, 500 and 1000 mg/kg body weight for 28 days. Mutation induction in the liver and glandular stomach was tested for, both results being negative [H20(FY2008)].

Since the TGR tests performed in mouse liver and glandular stomach were negative, the DNA damage detected in the comet test is considered to have been repaired in vivo*.* Thus, there is a high possibility that such DNA damage would not lead to mutation. In conclusion, there are no concerns that Food Red No. 105 induces genotoxicity (mutagenicity) in vivo.

## Discussion

The standard genotoxicity tests are carried out to detect gene mutation by Ames test using bacteria, and to detect chromosomal abnormalities by an in vitro chromosomal aberration test using cell culture and an in vivo micronucleus test using mice. Chromosomal abnormalities in chromosomal aberration tests are observed as morphological abnormalities in chromosomes during the interphase of cell division because damaged DNA is not normally replicated and the abnormalities persist. Such structural abnormalities are lethal for cells in many cases, and the majority of chromosomal abnormalities are not inherited by the next generation. Similarly, micronuclei in the micronucleus test also transiently appear in daughter cells after cell division, and disappear after the next cell division. Therefore, chromosomal abnormalities and micronuclei are indicators that DNA has been exposed to genotoxic substances, not a cause of cancer in cells. The fragmentation of DNA observed in the comet test is also transient, thus the comet test is also an indicator test. On the other hand, gene mutation is irreversible and permanent. Gene mutations arising in oncogenes or tumor suppressor genes have the possibility to cause cell transformation and initiate cancer-forming cells. Therefore, genetic mutation is a direct trigger of cancer, and it is highly correlated with carcinogenicity in rodents compared to other genotoxic end points [18], while the chromosome aberration test and micronucleus test have high false positive rates and low correlation with carcinogenicity tests [19].

The TGR test, which is an in vivo gene mutation test, is thus recommended when chemical substances have shown positive results in chromosome aberration tests, micronucleus tests, and comet tests. In particular, when comet and TGR tests are carried out on the same target tissue, if the results differ between the two, the results of the TGR tests should be given priority. The TGR test is also useful for follow-up of the same gene mutation test, the Ames test. A false positive reaction sometimes occurs in the Ames test because of bacteria-specific conditions such as drug metabolism, in vitro test-specific reactions using rat S9, as well as nonspecific reactions due to non-physiological conditions differing from the in vivo situation. Confirmation of an indication of mutagenicity with the Ames test by the TGR test in the living body is important on both scientific and safety grounds.

Among the 13 designated food additives covered in the Commentary section, eight items were positive for the Ames test, but the TGR test showed negative results for all of them. As a result, the possibility that these eight food additives exhibit genetic toxicity (especially mutagenicity, which is problematic for living bodies) is eliminated. This knowledge is important to ensure human safety. The TGR test took effect with publication of the OECD Guideline TG488 in 2011 and therefore was not available for implementation at the time of the Hayashi report (2000). We expect the safety of other food additives to be confirmed as TGR test results are accumulated.

## Postscript

In this report, we summarized the data for the most widely used substances in the classification of designated food additives in Japan. Currently we are summarizing the results of genotoxicity tests conducted at MHLW for existing food additives, a group of the next most widely used food additives, in the same way. The reports will be updated from time to time since additions and deletions of items are considered likely in the future.

## Additional file

Additional file 1: References Reports in Japanese. (DOCX 14 kb)

## Abbreviations

CA: Chromosomal aberration test
CHL: Chinese hamster lung
FSC: Food Safety Commission
FY: Fiscal year
MHLW: The Ministry of Health, Labour and Welfare
MN: Micronucleus test
NIHS: National Institute of Health Sciences
OECD: The Organisation for Economic Co-operation and Development
TGR: Transgenic rodent gene mutation assay
TMRL: Tokyo Metropolitan Research Laboratories of Public Health
