# Supplementary material for: Summarized data of genotoxicity tests for designated food additives in Japan
Source: Genes Environ. 2018 Dec 26;40:27. doi: 10.1186/s41021-018-0115-2 (PMC6311025; doi:10.1186/s41021-018-0115-2)
Supplement: Supplementary file 1 — References Reports in Japanese. (DOCX 14 kb) [file 41021_2018_115_MOESM1_ESM.docx]

**Additional file 1: References in Japanese**

A: Entrusted examinations by Ministry of Health and Welfare or MHLW

H6(FY1994), H11**(**FY1999) - H13(FY2001), H15(FY2003) - H18(FY2006), H20(FY2008) - H26(FY2014)

B: Risk Assessment Reports of Food Safety Commission, Accessed 24 Nov 2018

FSC1: http://www.fsc.go.jp/fsciis/evaluationDocument/show/kya20120402449

FSC2: http://www.fsc.go.jp/fsciis/evaluationDocument/show/kya20031020097

FSC3: http://www.fsc.go.jp/fsciis/evaluationDocument/show/kya20081030009

FSC4: (L-Ascorbic acid-2-glucoside)

FSC5: http://www.fsc.go.jp/fsciis/evaluationDocument/show/kya20141017263

FSC6: http://www.fsc.go.jp/fsciis/evaluationDocument/show/kya20031121192

FSC7: http://www.fsc.go.jp/fsciis/evaluationDocument/show/kya20151106466

FSC8: http://www.fsc.go.jp/fsciis/evaluationDocument/show/kya20111007037

FSC9: http://www.fsc.go.jp/fsciis/evaluationDocument/show/kya20120402447

FSC10: http://www.fsc.go.jp/fsciis/evaluationDocument/show/kya20110419009

FSC11: http://www.fsc.go.jp/fsciis/evaluationDocument/show/kya20050328692

FSC12: http://www.fsc.go.jp/fsciis/evaluationDocument/show/kya20050328693

FSC13: http://www.fsc.go.jp/fsciis/evaluationDocument/show/kya20081030006

FSC14: http://www.fsc.go.jp/fsciis/evaluationDocument/show/kya20031121105

FSC15: http://www.fsc.go.jp/fsciis/evaluationDocument/show/kya20071024003

FSC16: http://www.fsc.go.jp/fsciis/evaluationDocument/show/kya20110426025

FSC17: http://www.fsc.go.jp/fsciis/evaluationDocument/show/kya20031121106

FSC18: http://www.fsc.go.jp/fsciis/evaluationDocument/show/kya20100615442

FSC19: http://www.fsc.go.jp/fsciis/evaluationDocument/show/kya20170321219

FSC20: http://www.fsc.go.jp/fsciis/evaluationDocument/show/kya20081030002

FSC21: http://www.fsc.go.jp/fsciis/evaluationDocument/show/kya20170321219

FSC22: http://www.fsc.go.jp/fsciis/evaluationDocument/show/kya20110426020

FSC23: http://www.fsc.go.jp/fsciis/evaluationDocument/show/kya20121212725

FSC24: http://www.fsc.go.jp/fsciis/evaluationDocument/show/kya20081030004

FSC25: http://www.fsc.go.jp/fsciis/evaluationDocument/show/kya20140829220

FSC26: http://www.fsc.go.jp/fsciis/evaluationDocument/show/kya20050815003

FSC27: http://www.fsc.go.jp/fsciis/evaluationDocument/show/kya20060522001

FSC28: http://www.fsc.go.jp/fsciis/evaluationDocument/show/kya20110426023

FSC29: http://www.fsc.go.jp/fsciis/evaluationDocument/show/kya20150605367

FSC30: http://www.fsc.go.jp/fsciis/evaluationDocument/show/kya20080208003

FSC31: http://www.fsc.go.jp/fsciis/evaluationDocument/show/kya20080208004

FSC32: http://www.fsc.go.jp/fsciis/evaluationDocument/show/kya20080208005

FSC33: http://www.fsc.go.jp/fsciis/evaluationDocument/show/kya20100514404

FSC34: http://www.fsc.go.jp/fsciis/evaluationDocument/show/kya20081030001

FSC35: http://www.fsc.go.jp/fsciis/evaluationDocument/show/kya20031121102

FSC36: http://www.fsc.go.jp/fsciis/evaluationDocument/show/kya20071024007

FSC37: http://www.fsc.go.jp/fsciis/evaluationDocument/show/kya20081030003

FSC38: http://www.fsc.go.jp/fsciis/evaluationDocument/show/kya20031020168

FSC39: http://www.fsc.go.jp/fsciis/evaluationDocument/show/kya20070206002

FSC40: http://www.fsc.go.jp/fsciis/evaluationDocument/show/kya20071024001

FSC41: http://www.fsc.go.jp/fsciis/evaluationDocument/show/kya20081030005

FSC42: http://www.fsc.go.jp/fsciis/evaluationDocument/show/kya20130731245

FSC43: http://www.fsc.go.jp/fsciis/evaluationDocument/show/kya20040816098

FSC44: http://www.fsc.go.jp/fsciis/evaluationDocument/show/kya20100315346

FSC45: http://www.fsc.go.jp/fsciis/evaluationDocument/show/kya20120402448

FSC46: http://www.fsc.go.jp/fsciis/evaluationDocument/show/kya20100816499

FSC47: http://www.fsc.go.jp/fsciis/evaluationDocument/show/kya20100430396

FSC48: http://www.fsc.go.jp/fsciis/evaluationDocument/show/kya20100405375

FSC49: http://www.fsc.go.jp/fsciis/evaluationDocument/show/kya20110104671

FSC50: http://www.fsc.go.jp/fsciis/evaluationDocument/show/kya20090914002

FSC51: http://www.fsc.go.jp/fsciis/evaluationDocument/show/kya20071024004

FSC52: http://www.fsc.go.jp/fsciis/evaluationDocument/show/kya20110421514

FSC53: http://www.fsc.go.jp/fsciis/evaluationDocument/show/kya20031121108

FSC54: http://www.fsc.go.jp/fsciis/evaluationDocument/show/kya20081121002

FSC55: http://www.fsc.go.jp/fsciis/evaluationDocument/show/kya20081030007

FSC56: http://www.fsc.go.jp/fsciis/evaluationDocument/show/kya20050621001

FSC57: (Polyvinyl polypyrrolidone)

FSC58: http://www.fsc.go.jp/fsciis/evaluationDocument/show/kya20141105273

FSC59: http://www.fsc.go.jp/fsciis/evaluationDocument/show/kya20040526002
